# Assessing potential aquatic toxicity of airport runoff using physicochemical parameters and *Lemna gibba* and *Aliivibrio fischeri* bioassays

**DOI:** 10.1007/s11356-020-09848-0

**Published:** 2020-07-15

**Authors:** Olga C. Calvo, Gisela Quaglia, Anubhav Mohiley, Maria Cesarini, Andreas Fangmeier

**Affiliations:** 1 Institute of Landscape and Plant Ecology, University of Hohenheim, August-von-Hartmann- Str. 3, D-70599 Stuttgart, Germany; 2 Department of Environment, Ghent University, Coupure Links 653, B-9000 Ghent, Belgium; 3 Institute of Evolution & Ecology, University of Tübingen, Auf der Morgenstelle 5, D-72076 Tübingen, Germany

**Keywords:** Airport runoff, Deicers, Ecotoxicity, Bioassays, *Lemna gibba*, *Aliivibrio fischeri*

## Abstract

A critical problem derived from airport operations is the environmental impact of runoff water. Airport runoff includes a complex mixture of pollutants, e.g., from deicing agents, that may affect negatively natural water bodies. This study assesses the spatial and temporal aquatic ecotoxicity of runoff water and possible aeroplane drift in a German airport. Over winter 2012–2013, from November to May, water samples were collected within the airport and surrounding area. These samples were analyzed using traditional physicochemical analysis and biotests with two aquatic organisms from different trophic levels, *Lemna gibba* and *Aliivibrio fischeri.* Overall, the samples examined in this study were relatively non-toxic to the tested organisms. The physicochemical parameters were mainly influenced by the sampling period being higher in colder months. In contrast, the ecotoxicity was influenced by the sampling site. For sites within the airport, a high correlation between the physicochemical parameters (EC and TOC) and toxicity in *L. gibba* was found. These correlations were not evident in samples taken outside the airport or when *A. fischeri* was used as a bioindicator. However, a pronounced seasonality has been observed, linked to the coldest months with average inhibition values of 50% in *L. gibba* and 25% in *A. fischeri*, particularly in January. Both biotests yielded differing results; therefore, more biotests should be included. However, *L. gibba* showed a good response with this type of water samples to be included in future studies together with detailed chemical analysis. The present study provides data to assess the potential ecotoxicological effects of airport runoff affected by winter operations.

**Electronic supplementary material:**

The online version of this article (10.1007/s11356-020-09848-0) contains supplementary material, which is available to authorized users.

## Introduction

Air traveling is regarded as the most convenient method of traveling long distances. However, airport activities may pollute the air, water, and soil (Cancilla et al., 2003; Nunes et al., 2011). In this regard, runoff waters formed by rainwater, melted snow, and deposition of everyday activities, e.g., refueling, aircraft and vehicles repairing/maintenance, de-/anti-icing operations, and chemical weed control, may pose a risk when they enter the environment (Corsi et al., 2009, 2001; Fisher et al., 1995; Sulej et al., 2014).

In cold climates, pavement deicer materials (PDMs) and aircraft deicer/anti-icing agents (ADAFs) are used to ensure the safe takeoff and landing of aircrafts (Freeman et al., 2015). Airplanes are usually sprayed with a mixture consisting of a chemical deicing fluid mainly based on glycol having other additives consisting of corrosion inhibitors, thickeners, surfactants, antifoaming agents, and dyes (Johnson, 2012). The formulation of these additives is a proprietary mixture differing among manufacturers.

ADAFs are usually applied at specific airport locations, commonly equipped by draining units that collect spent fluids and runoff water to collection systems. Even where deicing wastewater is drained to a dedicated runoff collection system, wind drift, jet blast, and absorption into pavements or soil may contribute to the dispersal of deicing products and migration into nearby surface waters such as lakes and streams (Nunes et al., 2011; Shi et al., 2017). Approximately, 75–80% of deicing fluids were found to deposit immediately on the pavement of the deicing area, while the remaining 15–20% was lost during take-off or taxiing (Switzenbaum et al., 2001) with a possible impact on the environment.

The composition of contaminants in the airport runoff may change because of the activities carried out at the airport, the time of the year, and also by the weather conditions (cold days, snowfall) (Freeman et al., 2015; Jia et al., 2018). Besides deicing products, airport runoff could also include a wide variety of chemicals and pathogens (Corsi et al., 2006b; Sulej-Suchomska et al., 2016; Sulej et al., 2012). Chemical analysis of airport runoff water samples aims to identify and quantify potentially harmful chemicals that may affect natural water bodies. However, these analyses do not allow the assessment of ecotoxicological impacts of pollutant mixtures and neither the possible interaction among them (Cancilla et al., 2003; Pillard, 1995). Bioassays are a valuable option for assessing the ecotoxicological impacts of chemicals in complex water samples (Bori et al., 2016; Hongxia et al., 2004). *Lemna gibba* (Brain et al., 2004; Greenberg et al., 1992; Mihaich et al., 2009; Nunes et al., 2014) and *Aliivibrio fischeri* bioassays have been used in ecotoxicological studies to investigate aquatic toxicity of a variety of pollutants (Reemtsma et al., 1999). Aquatic toxicity has been observed in water receiving ADAFs using bioassays, such as *Aliivibrio fischeri* (Corsi et al., 2009, 2006a, 2001; Mohiley et al., 2015). *L. gibba* sp. has been used to study environmental pollution from industrial wastewater by using growth parameters and biochemical assays (Radić et al., 2010). However, to the best of our knowledge, as yet the *Lemna* sp. biotest has not been applied to airport runoff water samples.

The collection and treatment of airport runoff with deicing contaminants can be costly and present challenges to the airport operation (Shi et al., 2017). To investigate how the intense use of deicers influences the airport runoff water and the waterbodies in the vicinity of an airport, a case study was performed in an airport in Germany. The impact of the runoff on the surroundings of airport has not yet been assessed. This case study is a first attempt to provide ecotoxicological data for the assessment of the environmental impact of airport runoff in two different directions: (a) the direct effect due to the airport runoff (e.g. containing deicers) and (b) the indirect effect through airplane drift during landing or taxing on waterbodies in the surroundings areas. The present study focuses on (1) temporal and spatial assessment of airport runoff water using bioassays and physicochemical analysis, and (2) on whether relationships exist between the water chemistry and the ecotoxicity data.

## Material and methods

### Site description

**Fig. 1:**
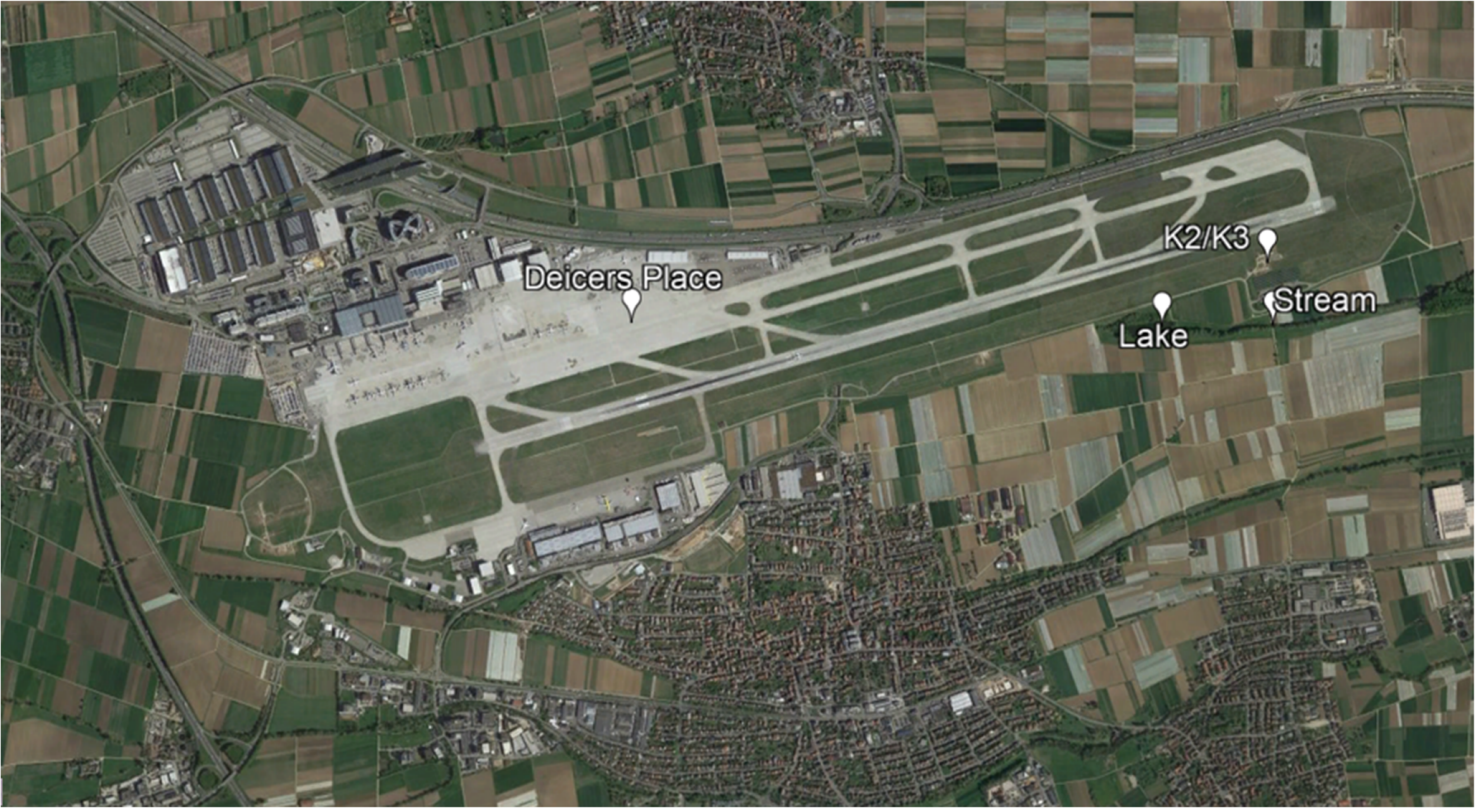
Localization of sampling sites at Stuttgart Airport (Google Landsat/Copernicus, 2017)

The airport (48° 41′ 24″ N 009° 13 ′19 ″E) is located in Stuttgart (Germany), 13 km SW from the city center at an altitude of 389 m. The airport runoff water is collected from the aircraft’s landing area and led to the internal wastewater treatment plant (WWTP). However, surface water in the vicinity of the airport is influenced by the airport, the surrounding agricultural areas, parking lots, and various roads including a major highway (Fig. 1).

The climate in the investigated area is predominantly temperate oceanic. The monthly average precipitation ranges from 40 mm in February to 93 mm in June, and the monthly average temperature ranges from 1.4 °C in January to 19.1 °C in July. The precipitation in 2012 and 2013 was 631 and 790 mm, respectively (LTZ, 2020) (for more details, see supplementary material Fig. A7).

Deicing of aircrafts takes place at special parking positions (Deicing Place) designed to capture deicers dripping from aircraft and transport them by truck to the WWTP. Part of the collected wastewater is pretreated within the wastewater network system of the airport, composed of 250 km of pipes, canals, tanks, and treatment facilities. When deicing activities are operating, the rainwater is channeled, collected, and treated according to the TOC values in three separate underground chambers for weak, moderate (K2), and heavily (K3) polluted water. Wastewater is collected in K3, and without any previous treatment is conducted to K2. In K2 oxygen and nutrients (N and P) are supplied to promote bacterial growth (fluidized-bed reactor) and limit TOC before the effluent can be conveyed to the WWTP of the villages Plieningen and Filderstadt-Sielmingen.

According to the information provided by the company managing the Stuttgart airport, three different aircraft deicers formulations have been used during the winter months 2012–2013. All these products are manufactured by a single company and fall within the Type I, Type II, and Type IV ADAF. All these product formulations are triazole-free. PDMs have also been used at the Stuttgart airport during the same period, both as solid and fluid materials (Flughafen Stuttgart, personal communication) (for more details, see Fig. A6 and Table A6 in supplementary material).

### Sample collection

The samples were collected monthly from November 2012 to May 2013. The physicochemical and ecotoxicological parameters were measured in the water samples collected corresponding to five sites with different deicing concentrations from the Stuttgart airport and water surfaces in its immediate vicinity (Fig. 1). In total, the potential toxicity of 51 samples representing 7 months and 5 sites was collected and analyzed (Table 1).

**Table 1:** Available data from runoff samples collected at different sites of an airport during the winter months 2012–2013

	Deicing	Lake	Stream	K2	K3
Nov	X	X	X		
Dec	X *	X *	X *	X *	X *
Jan	X *	X *	X *	X *	X *
Feb	X *	X	X	X	X
Mar	X	X *	X *	X *	X *
Apr	X*	X *	X *	X *	X *
May	X	X	X		

X, *Lemna*; *Aliivibrio

Water samples were collected manually in 1000 mL water-tight polyethylene bottles with a plastic scoop. Before use, the material was rinsed with the sampled water. The samples were transported within 1 h to the laboratory and stored at − 20 °C in the dark until further analysis. The samples were defreezed at different time points, just before performing each biotest or physico-chemical analyses. No chemicals were added to preserve the samples. Some collected water samples were contaminated with solids, but the sediments were not taken for the toxicity test. Before performing the experiment, samples were thawed at room temperature. The control consisted of deionized water, which was stored in the same plastic bottles and used in the same way as the collected runoff water samples, to control possible negative effects of plastic.

### Physicochemical assessments

The collected water samples were subjected to physicochemical analysis. The scope of the analysis included the determination of pH and electrical conductivity (EC) using potentiometric and conductometric methods, respectively. Additionally, TOC was determined by the airport laboratory. BOD requires a longer time to be analyzed. On the contrary, TOC values can be obtained in hours. Hence, for the operation of the wastewater of the airport, a TOC allows faster management (Assmann et al., 2017). It has been reported that both parameters are correlated (Assmann et al., 2017; Dubber and Gray, 2010).

### Ecotoxicity assessments

In order to evaluate the spatial and temporal variation of the ecotoxicological potential of airport runoff water samples, two aquatic biotests, based on the results obtained from a previous study dealing with ADAFs and wastewater samples containing these compounds, have been implemented (Mohiley et al., 2015). The quality criteria set by the guidelines concerning the procedure for each bioassay were met.

### *Lemna* sp. growth inhibition test

The first biotest conducted was the *Lemna* sp. growth inhibition test according to OECD guideline 221 (OECD, 2006). The plant cultures were maintained in the AAP growth medium (OECD, 2006) in a controlled climate chamber (Fitotron Model S.G.C. 120, Weiss Gallenkamp, UK) with an adjusted temperature of 24 °C, under continuous illumination at an intensity of 100 ± 15 μmol^−2^ s^−1^ using fluorescent lamps (Philips, Mater TL-D 36 W/840 OF Poland). To test the effects of wastewater samples, twelve healthy fronds were transferred into glass beakers (250 mL, 6.5 cm Ø, VWR, Germany) filled with 150 mL of a test solution. A semi-static 7-day test was performed, changing the growth medium on the fourth day. The beakers were randomized within the chamber every second day. Two endpoints were measured, the frond number counted visually, and the frond area determined by using the ImageJ software (NIH, USA).

Each sample was tested with three replicates and four dilutions 1/4, 1/8, 1/16, and 1/32 corresponding to the concentrations of 250 mL L^−1^, 125 mL L^−1^, 62.5 mL L^−1^, and 31.25 mL L^−1^. Test concentrations including control (deionized water) were prepared by diluting the samples with the AAP growth medium (OECD, 2006).

### Light inhibition bioassay of *Aliivibrio fischeri*

Acute toxicity to the luminescent bacteria *A. fischeri* (NRRL B-11177) was assessed following ISO guideline 11348-2 (DIN EN ISO, 2007). This bacterial luminescence test with the bacteria *A. fischeri* is frequently chosen as the first step in a battery of toxicity biotests to check the toxicity of wastewater samples from industries, since it is rapid and cost-effective method (Reemtsma et al., 1999).

Since *A. fischeri* is a marine bacterium, the osmotic pressure of samples was adjusted to a conductivity of 32 mS cm^−1^ with a 2% NaCl solution (Merck KG, Germany). The luminescence inhibition test with liquid-dried bacteria stored at − 20 °C was performed according to the instructions of the product BioFix (Macherey-Nagel, Düren, Germany). The inhibition of the bacteria natural light emission was measured against a control (2% NaCl) using a luminometer (Lumistox 300, Hach-Lange, Germany) on two technical replicates after 30 min of exposure of the bacteria to the sample (1:1) at 15 °C in glass cuvettes. For this experiment, we had three replicates. Tests were carried out on dilutions of 31.25 mL L^−1^, 62.5 mL L^−1^, 125 mL L^−1^, and 250 mL L^−1^ of collected samples. Test concentrations and control were prepared by diluting the samples with 2% NaCl solution.

### Statistical analyses

Statistical analyses were performed with the R programming language 3.0.1 (Ritz et al., 2015). The toxicity of a sample was quantified by the effective concentration values (EC_10_ / EC_50_) determined by fitting the appropriate dose-response curve. The regression curves were modeled using a three-parameter log-logistic model or a linear/cubic selecting form AIC (Akaike Information Criterion) parameter using the *drc* package (Appendix E). The residuals were evaluated after the model selection.

Two-way ANOVA was performed to compare the toxicity values at five sampling locations during different months. Sampling locations and sampling periods served as independent variables and the measured endpoints (frond biomass in *L. gibba* and luminescence inhibition in *A. fisheri*) as dependent variables. The significance level was set at *p* ≤ 0.05. In case of a lack of homogeneity of variances and normality, the non-parametric Kruskal-Wallis test was used.

Principal component analysis (PCA) was performed to identify the possible connection of physicochemical parameters of airport runoff water samples with their ecotoxicological effects. The PCA reduced the 6 months, 3 sampling places, and 2 bioassays to two principal components with eigenvalues. The sites K2 and K3 from November and May were not included in the calculations of the PCA because the chambers were empty and had no samples to be collected. Moreover, Pearson correlation coefficients were calculated to find potential significant relationships between the results of toxicity tests and physicochemical parameters. To ensure that the results of the ecotoxicological and physicochemical analysis increase in the similar way, a toxic value (TV10) expressed as the inverse of EC_10_ was chosen (TV10 = 1/EC_10_). A higher toxic value indicates a higher negative effect of a wastewater sample on the organism.

## Results and discussion

In this work, the potential toxic effect of airport runoff was studied using two different biotests, the *Lemna* sp. growth inhibition test and the bacterial luminescence test. Results on the toxicity criteria, half-maximal effective concentration (EC_50_), or 10% effective concentration (EC_10_) obtained from both bioassays are summarized in Appendix B (Tables A4-A5). To permit a comparison of the sensitivity of tested organisms, the values of EC_10_ were determined.

Runoff samples tested in this study were relatively non-toxic to the tested organisms, *L. gibba* and *A. fischeri*. We observed significant differences in ecotoxicity depending on the site. Besides the determination of the potential ecotoxicity of runoff samples, we also measured the main physicochemical parameters (pH, conductivity, and TOC). Similar to Bojarczuk et al. (2018), seasonal changes in the studied parameters were more evident in the case of physicochemical parameters rather than for the microbiological indicators of water quality. While pH differed depending on the site (*p* < 0.01), conductivity differed depending on the sampling period (*p* < 0.01). We observed significant differences of TOC depending on both, the site (*p* < 0.001) and the sampling period (*p* < 0.01) (Table 2).

**Table 2:** Two-way ANOVA results for ecotoxicological (EC_10_) and physicochemical values from samples collected on seven occasions (Nov-May) and at five different sites (Deicing Place, K2, K3, lake, stream) during winter 2012–2013 at a regional airport

	Site	Period
EC_10_ FN [mL L^−1^]	**	–
EC_10_ FA [mL L^−1^]	**	–
EC_10_ BIO [mL L^−1^]	*	–
pH	**	–
EC [μS cm^−1^]	–	**
TOC [mg L^−1^]	***	**

*−*p ≤* 0.05, **p ≤ 0.01, ***p ≤ 0.001,- not significant. *FN* frond number of *L. gibba*, *FA* frond area of *L. gibba*, *BIO* bioluminescence of *A. fischeri*, *EC* electrical conductivity, *TOC* total organic carbon

### Physicochemical characteristics of airport runoff water

The physicochemical characteristics of runoff samples collected in the winter period 2012–2013 at the Stuttgart airport are shown in Appendix C (Table A6). Results of the two-way ANOVA of each physicochemical parameter are summarized in Table 2.

In this study, the selected sites were differentiated into two groups, the runoff catchment points inside the airport with higher conductivity values, in K2 and K3 reaching mean values up to 5200 μS cm^−1^, which are lower than the reported values for industrial wastewater (10,000 μS cm^−1^) (American Public Health Association, 1999) in one group. While in another group, the natural water surfaces outside the airport presenting lower conductivity values around 1000 μS cm^−1^ which were within the environmental quality standards in the German Surface Water Regulation and the range of normal values for calcareous water (600–1200 μS cm^−1^) (BMJV, 2016) (Figs. 4e and 5a). Conductivity values from samples collected in the lake were higher than in the stream; this could result from an accumulation of salts from a nearby road, neighbor agricultural soils, or/and from a restricted outflow in the lake.

However, the conductivity of runoff samples was mainly influenced by the sampling period (Table 2). Conductivity varied during the winter months, increasing from the initial sampling month November (361 μS cm^−1^ as average), reaching the highest values in February (2468 μS cm^−1^ as average) and decreasing to lower values in May (847 μS cm^−1^ as average) (Fig. 4b). The respective weather event that necessitates deicing activities, its duration, the time elapsed since the last rain event, and the amount of precipitation are factors known to strongly influence the concentration of different contaminants in runoff (Corsi et al., 2009). In fact, conductivity values were significantly negatively correlated with the amount of daily precipitation (before collecting samples), especially in sample points outside the airport (*r* = − 0.9**), but not with the mean monthly values (Table 3). Similarly, Bojarczuk et al. (2018) reported that the higher the water flow, the lower was the conductivity concentration in a river. By contrast, Jia et al. (2018) found a positive correlation of conductivity with precipitation; however, this correlation was calculated only during warmer months.

**Table 3:** Pearson’s product-moment correlation coefficient between physicochemical parameters and ecotoxicological values (toxic value TV10), together with the total quantity of applied deicers (VOLdeicers) and daily precipitation before sampling (Pdaily)

Site	Feature	pH	EC	TOC	TV10 (FN)	TV10 (FA)	TV10 (BIO)
Deicing Place	pH	1					
	EC	–	1				
	TOC	0.83*	0.94**	1			
	TV10 (FN)	–	0.85*	0.95***	1		
	TV10 (FA)	–	0.85*	0.95***	0.99***	1	
	TV10 (BIO)	–	–	–	–	–	1
	VOLdeicers	–	–	–	–	–	–
	Pdaily	–	–	–	–	–	–
K2	pH	1					
	EC	–	1				
	TOC	–	0.87**	1			
	TV10 (FN)	–	0.98***	0.84*	1		
	TV10 (FA)	–	–	0.90**	0.79*	1	
	TV10 (BIO)	−0.98*	–	–	–	0.91*	1
	VOLdeicers	–	–	–	–	–	–
	Pdaily	–	–	–	–	–	–
K3	pH	1					
	EC	–	1				
	TOC	–	0.96***	1			
	TV10 (FN)	–	0.77*	0.82*	1		
	TV10 (FA)	–	0.83*	0.87**	0.99***	1	
	TV10 (BIO)	–	0.96**	–	–	–	1
	VOLdeicers	0.90*	–	–	–	–	–
	Pdaily	–	–	–	–	–	–
Lake	pH	1					
	EC	–	1				
	TOC	–	–	1			
	TV10 (FN)	–	–	–	1		
	TV10 (FA)	−0.85*	–	–	0.81*	1	
	TV10 (BIO)	–	–	–	–	–	1
	VOLdeicers	0.77*	–	–	–	–	–
	Pdaily	0.88**	−0.86**	–	–	–	–
Stream	EC	–	1				
	TOC	–	−0.88**	1			
	TV10 (FN)	–	–	0.95***	1		
	TV10 (FA)	–	–	–	–	1	
	TV10 (BIO)	0.99**	–	–	–	–	1
	VOLdeicers	–	–	–	–	–	–
	Pdaily	–	−0.87**	0.96***	0.90**	–	–

*−*p ≤* 0.05, ***p ≤ 0.01, ***p ≤ 0.001, - not significant. *EC* electrical conductivity, *TOC* total organic carbon, *TV10* toxic value (=1/EC_10_), *FN* frond number, *FA* frond area, *BIO* bioluminescence

Interestingly, conductivity is not correlated with the volume of applied deicers (concerning only ADAFs) at any location. Indeed, Deicing Place, where the maximal amounts of ADAFs are present, showed the lowest conductivity values of sites inside the airport (Appendix C, Table A7). These values contrasted with the higher conductivity values measured in the runoff collection basins (K2 and K3), indicating a presence of salts from other sources such as PDMs (e.g., sodium acetate and sodium formate). Similarly, it has been reported that the highest values for conductivity were obtained in samples from the highway runoff collected in January and February due to the accumulation of salts in snow coversed roads in winter (Asensio et al., 2017; Szklarek et al., 2015; Waara and Färm, 2008).

On the other hand, the pH of the collected samples was not influenced by the sampling period but by the location (Table 2). The pH of runoff samples in the colder months increased dramatically in the three areas inside the airport; especially K3 presented samples with significant higher alkalinity (pH between 9 and 9.5) in all months (Fig. 4a). Actually, pH was significantly positive correlated (*r* = 0.90*) with the volume of applied deicers (Table 3). Similarly, a pH increase in the drainage catchment has been reported in a Swedish Airport (up to 9.3) (Jia et al., 2018) and two international airports (up to 8.6) (Fisher et al., 1995; Freeman, 2016). Many aquatic organisms have a relatively low tolerance to variations in pH. Aquatic plants could be massively damaged by the introduction of large quantities of alkaline wastewater. The high pH value decreased in the next catchment area (K2). It should be mentioned that the runoff of this airport is not released into the environment but is transported to the next WWTP.

By contrast, pH values in the studied water surfaces outside the airport remained in the neutral range (7–8.5) (Fig. 4d). These values are within environmental quality standards in the German Surface Water Regulation (BMJV, 2016).

Airports use TOC as a general parameter to describe the presumed toxicity of wastewater samples. TOC was significantly influenced by both sampling period and location (Table 2). Similar to conductivity, TOC increased during the colder months presenting a peak in January (Fig. 4c). This seasonal effect on TOC could be due to a decreased microbial activity at lower temperatures as it has also been reported by Regnery et al. (2015).

Runoff storage sites inside the airport presented higher TOC values, up to 1500 mg L^−1^ in K3, 500 mg L^−1^ in K2, and 300 mg L^−1^ in Deicing Place. These values are similar to the values observed in an airport with low capacity of passenger movement (Sulej et al., 2014) and much lower than the values that would be observed in an airport with high capacity of passenger movement (up to 22,000 mg L^−1^) which were associated with oil derivatives and polycyclic aromatic hydrocarbons (PAH) emitted during combustion and uncontrolled spillage of aviation fuels and lubricants (Sulej et al., 2014). Our study indicates that ADAFs are not the main component causing higher TOC values; PAH emissions from uncontrolled fuel/oil spills could also play a role in the higher TOC values found.

By contrast, both water surfaces outside the airport (lake and stream) in general presented organic carbon contents close to zero (Fig. 4f). They were following the limit values from the German Surface Water Regulation (< 7 mg L^−1^) (BMJV, 2016). Similar low TOC values (between 4 and 10 mg L^−1^) have been reported in a river due to the riverbank filtration (Regnery et al., 2015). However, some samples such as from lake in January (31 mg L^−1^) and from the stream in November (13 mg L^−1^) were slightly higher than these limits. Moreover, TOC values from water samples collected in the stream in November correlated with the daily precipitation (Table 3), indicating possible incorporation of runoff from agricultural fields, a parking place, or a roadway in the vicinity.

In our study, TOC was significantly positively correlated with conductivity in Deicing Place (*r* = 0.94**), while in the stream outside the airport TOC was significantly negatively correlated with conductivity (*r* = − 0.88**) (Table 3). Moreover, we observed a significant positive correlation between TOC and pH in Deicing Place (*r* = 0.83*), probably due to the alkalinity of ADAF Type I (pH = 8–9.5) and the PDM Na-Formate (pH = 9).

### Ecotoxicity assessment of airport runoff water

In general, the toxicity from both biotests followed a similar trend for runoff from inside the airport. In contrast, both biotests showed different ecotoxicity for runoff from environmental samples outside the airport. The variation in toxicity among species may imply that some pollutants are more toxic to one species than to others. For instance, a study dealing with PDMs, *A. fischeri* showed opposite behavior than five other biotests (Corsi et al., 2009).

### Lemna Growth Inhibition Test

In the Deicing Place, lake, and stream, the Lemna Growth Inhibition Test was conducted from November–May, and in K2 and K3 from December–April. The EC_10_ values of runoff samples for frond number and frond area ranged between 13 and 323 mL L^−1^ and 9 and 379 mL L^−1^, respectively (Appendix B, Table A4b). At none of the concentrations applied, fronds showed any chlorotic effects.

In most samples, we could not calculate the half-maximal effective concentration (EC_50_) for the studied endpoints, frond number and frond area. Similar low toxicity was detected with *L. minor* in the presence of highway runoff (Waara and Färm, 2008). Therefore, we used EC_10_ for the comparison of runoff samples (Appendix B, Table A4).

The two endpoints (frond area and frond number) measured on *L. gibba* presented similar toxicity (EC_10_) values in all runoff collection points, except in the stream (Appendix B, Table A4). Indeed, the Pearson correlation coefficient between these two endpoints was very high in all locations (*r* = 0.79* to 0.99***), except in samples collected in the stream (Table 3).

**Fig. 2:**
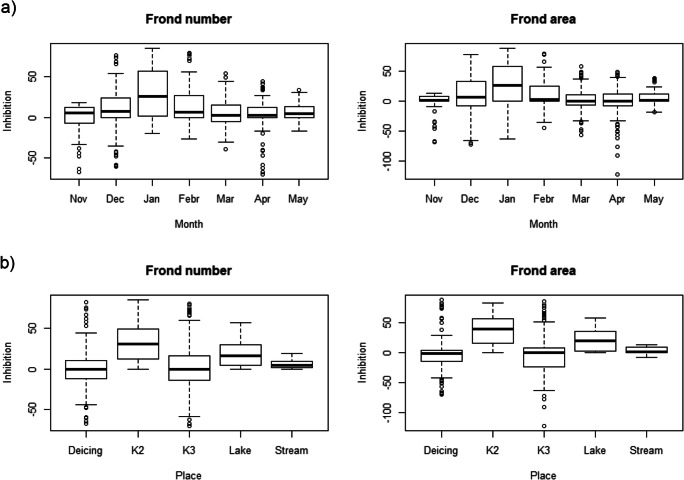
Inhibition [%] of frond number and frond area of *L. gibba* from all tested dilutions of runoff samples collected **a** in seven winter months (Nov–May) and **b** at five different locations (Deicing place, K2, K3, Lake, Stream) during winter 2012–2013 at a regional airport. Centerline shows the median; box limits indicate the 25th and 75th percentiles. Whiskers represent the lowest and highest values

Based on the results of ANOVA, the toxicity of collected samples towards *L. gibba* was mainly influenced by the sampling site (Table 2). While stream water samples showed no inhibitory effects on *L. gibba*, K2 samples showed the highest inhibitory effects reaching up to 50% inhibition levels (Fig. 2b) with mean EC_10 (FA)_ values of 9–12 mL L^−1^ in the winter months (December–February) (Appendix B, Fig. A4b). Similar to K2, lake water sample EC_10 (FA)_ values were 18 and 27 mL L^−1^ in December and January, respectively (Appendix B, Fig. A4b). The Deicing Place and K3 showed the highest variability in results concerning *L. gibba* growth, ranging from a toxicity effect in January to a fertilization effect in the rest of the studied months (Fig. 2b, Appendix B Table A4b). Remarkably, similar water sampling and testing performed at the same sites (Deicing Place and K3) of the airport in summer (June 2013) showed relative low toxicity to *L. gibba* (Mohiley et al., 2015). This is an indirect indication of the role of winter airport operations and prompts further research towards a better understanding of the aquatic toxicity of the runoff mixture. Indeed, this airport uses in winter several ADAFs (different types and quantities) according to the different weather conditions (Appendix A Figs. A6, A7). It has been suggested that toxicity of surfactants such as the alcohol ethoxylate may account for a portion of the observed toxicity in ADAFs formulations (Corsi et al., 2006c). We should also take into consideration that during warmer periods, the detrimental effect of xenobiotics, including ADAFs, present in wastewater could be positively counterbalanced by other factors such as a higher bacterial degradation rate of the toxic substances due to increased temperatures.

Despite the limited number of samples tested, a distinct seasonality could be observed in the response pattern of the two endpoints in the water samples of the underground chambers (K2/K3). Whenever significant effects were detected, they were distinctly associated with the coldest months when deicers and anti-icers were used. Exposure of *L. gibba* to increasing concentrations of runoff collected from the winter months (December–February) caused a significant dose-dependent reduction of frond number/area, especially in January reaching mean values of 50% inhibition (Fig. 2a) with the lowest EC_10_ values, 13 and 10 mL L^−1^ for frond number and frond area, respectively (Appendix B Fig. A4b). No toxic response was observed in samples collected in spring (April, May) in the wastewater reservoirs (Fig. 2a). This result is in agreement with Corsi et al. (2001), who tested runoff water samples from the General Mitchel International Airport (Milwaukee, Wisconsin). These authors found that samples collected during winter storm events had higher acute toxicity for *Pimephelas promelas* and *Ceriodaphnia dubia* than samples taken in summer. On the other hand, in all months, lower concentrations of runoff showed a stimulating effect on *L. gibba* growth (Fig. 2a).

As an important result of our work, we could establish for the first time, a dose-response relationship between *L. gibba* growth rates and the airport runoff. According to the lowest EC_10_ values found at the individual sites, the sensitivity of *L. gibba* towards runoff water was in the following ascending order: Stream < Lake < K3 < K2 < Deicing Place. It is important to add that obtained results only concern the Stuttgart airport and should be verified at different airports and on longer time scales.

### Bacterial luminescence test

Bacterial luminescence test was conducted in December, January, March, and April in the five studied areas. No EC_50_ values could be calculated for the bioluminescence test. Thus, we conclude that no acute toxicity occurred with the tested runoff samples under the conditions defined by the guideline. A lack of toxicity on *A. fischeri* has also been reported in experiments performed with ADAFs (Type I and Type IV) (Corsi et al., 2006c; Mohiley et al., 2015). Therefore, in this study, only EC_10_ values were calculated and used for comparison. The bioluminescence EC_10_ values of runoff samples ranged between 20 and 236 mL L^−1^ (Appendix B, Table A5).

**Fig. 3:**
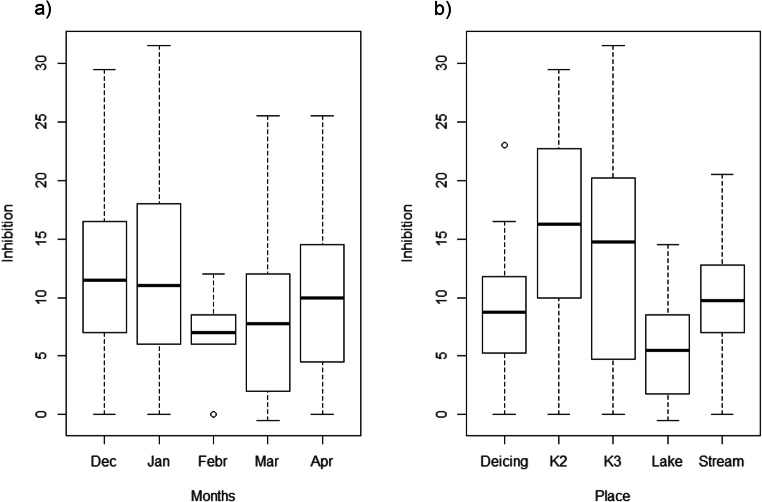
Inhibition [%] of *Aliivibrio fischeri* from all tested dilutions of all runoff samples collected **a** in five winter months (Dec–Apr) and **b** at five different locations (Deicing place, K2, K3, Lake, Stream) during winter 2012–2013 at a regional airport. Centerline shows the median; box limits indicate the 25th and 75th percentiles. Whiskers represent the lowest and highest values

According to the results of ANOVA, the toxicity of the collected samples for *A. fischeri* was influenced by the site, but not by the sampling period (Table 2). Runoff water samples collected inside the airport runoff catchment areas, K2 and K3, showed inhibition mean values of the bioluminescence between 20 and 25% (Fig. 3b) with the lowest EC_10_ values around 21 mL L^−1^ in the winter months December and January (Fig. 3a, Appendix B, Table A5). Similar inhibition values have been reported from highway runoff, and they were classified as non-toxic (Waara and Färm, 2008). This relatively low toxicity includes the effect of pH, which was not adjusted, and which has been considered to influence the toxicity response of *A. fischeri* (Ranke et al., 2004). Therefore, the inhibition caused by different compounds of airport runoff water samples should be even lower. In this study, we did not adjust pH because it has been reported that an increase in pH value would lead to a change in the original contaminant load in the environmental sample (Fomin et al., 2003). The underground chambers (K2/K3) collect water flooding from the airways and taxiways as well as from other airport areas, so it may be expected that the composition of water at this site may be more heterogeneous. Besides water contaminated by ADAFs dripping off onto the airfields, PDMs, such as K-formate or Na-formate, as well as metals or other persistent or unknown contaminants released by different sources (i.e., fuel, tire debris, deicers, tensides from detergents, pesticides, paints), could be present. In our study, it is not possible to discriminate which contaminant may be responsible for the toxic effects. Potassium formate has been reported to affect the growth of propylene glycol (PG) microbial degraders (Biró et al., 2014). On the other hand, the toxicity of PDMs on *A. fischeri*, contrarily to other bioindicators, has been reported to be driven primarily by its additives rather than acetates/formates (Corsi et al., 2009).

**Fig. 4:**
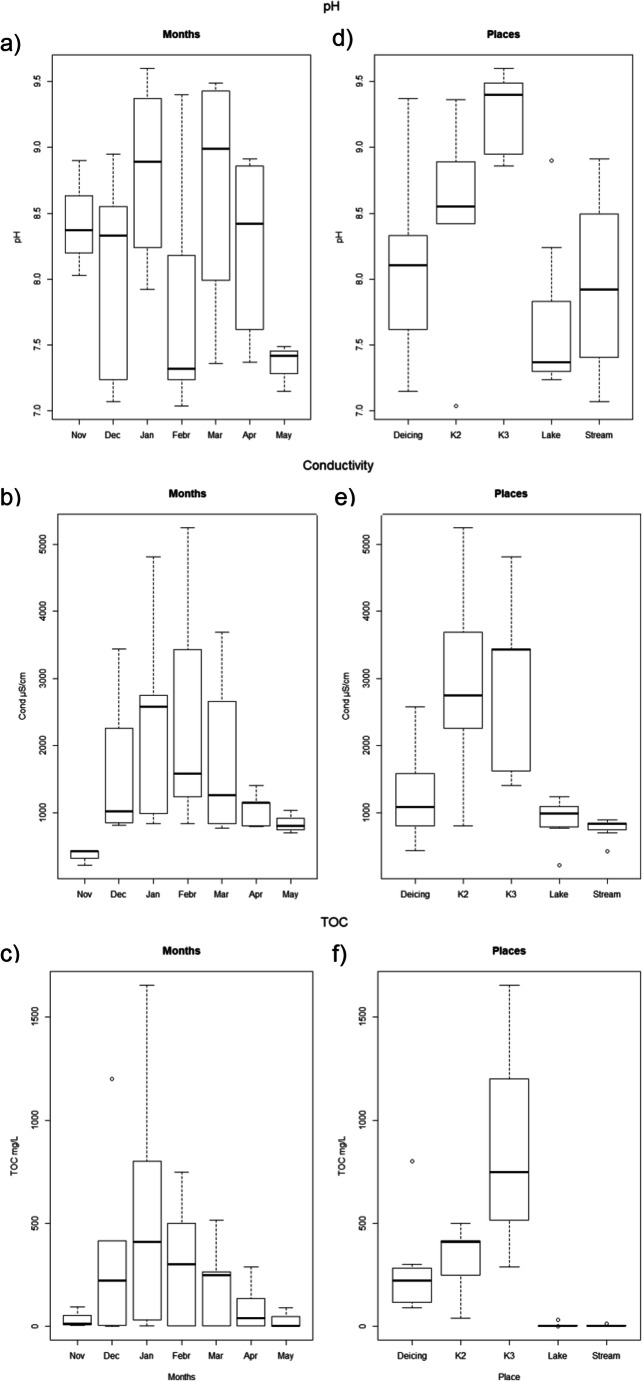
Physicochemical properties (pH, conductivity, TOC) of water samples collected during the winter months 2012–2013 (**a**–**c**), at different sites of a regional airport (**d**–**f**). Centerline shows the median; box limits indicate the 25th and 75th percentiles. Whiskers represent the lowest and highest values. *Conductivity* electrical conductivity, *TOC* total organic carbon

**Fig. 5:**
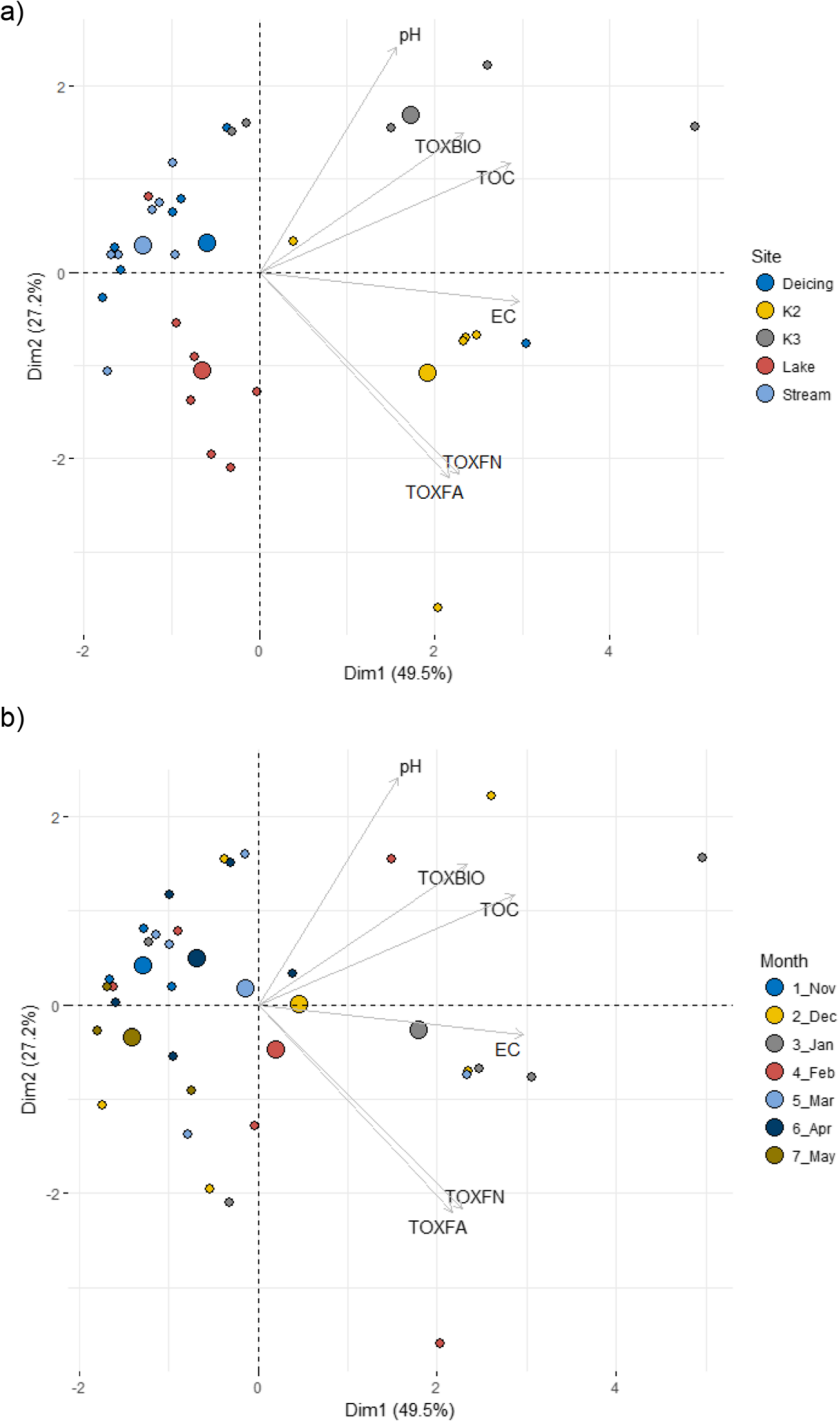
PCA-biplot showing the projections of the variables (EC, pH, TOC, TOXBIO, TOXFN, TOXFA) according to **a** the site and **b** the month. Vectors represent the strength and direction of environmental data. *TOX* toxic value TV10 (=1/EC_10_) of *FN* frond number and *FA* frond area of *L. gibba* and *BIO* bioluminescence *of A. fischeri*, *EC* electrical conductivity, *TOC* total organic carbon

*A. fischeri* has been shown to be the species with the highest susceptibility to some kinds of BTs (Molins-Delgado et al., 2015). In this study, the Deicing Place was presumably the area most directly affected by the deicers as most aircraft winter operations are carried out there. However, the fact that most runoff samples at the Deicing Place showed the lowest apparent toxicity among all places located inside the airport points to a lack of toxic compounds (such as BTs) contained in the additives, the formulation of which is confidential. In fact, the lack of BTs in the ADAFs applied at Stuttgart airport has been confirmed by the manufacturer (personal communication). Other possible input sources of BTs could be deposition from a road (Kiss and Fries, 2009).

On the other hand, Deicing Place together with samples collected outside the airport showed even lower mean inhibition values (12%). According to the lowest EC_10_ values found at each site, the hierarchy of the levels of sensitivity of *A. fischeri* towards runoff water was in the ascending order: Lake < Stream < Deicing Place < K3 < K2.

In this study, *A. fischeri* showed the lowest sensitivity. This low sensitivity is rather surprising since the *A. fischeri* luminescent test is susceptible to many different compounds (Kaiser and Palabrica, 1991) and widely accepted as a good indicator of the environmental impact of certain wastes and leachates produced by human activity (Roig et al., 2012; Zhou et al., 2017). Lower toxicity values have also been obtained in runoff samples from a waste landfill (Melnyk et al., 2014) and hydrocarbon-contaminated soils (Bori et al., 2016).

### Relationships between physicochemical and biological parameters

A principal component analysis (PCA) was performed for a graphical presentation of the obtained results and relationships between the physicochemical parameters of runoff and their potential role explaining the ecotoxicological effects (Fig. 5, Appendix D Fig. A8). The first two components allow interpreting approximately 77% (50% Dim1 and 27% Dim2, respectively) of the variance of the data. The first PCA component (Dim1) separated the coldest months (December and January) from the warmer months (November and May) (Fig. 5b). Moreover, this first component separated the runoff samples collected in different sedimentation tanks inside the airport (K2 and K3) from the environmental water samples located outside the airport, together with the Deicing Place (Fig. 5a).

In the present study, the physicochemical parameters contributed to explaining the ecotoxicological effects of airport runoff water in different ways. The traditionally physicochemical parameters correlated with all measured biological endpoints in the internal runoff, but not with water samples collected in the vicinity of the airport (Fig. 5a, Table 3). Similarly, it has also been reported that the chemical evaluation of samples does not always correspond to the toxic effects towards the bioindicator (Melnyk et al., 2014; Mendonça et al., 2009).

In the catchment tanks inside the airport, toxicity values of *A. fischeri* were positively correlated with conductivity in only one site (K3) (*r* = 0.96**). Toxicity values found in *L. gibba* were positively correlated with conductivity (*r* = 0.77* to 0.98***) and TOC (*r* = 0.82* to 0.95***) parameters, these two parameters being significantly positively highly correlated (Table 3). These results indicate the validity of TOC, as an easily detectable parameter to predict the potential ecotoxicological impact of airport runoff on plants, but not on bacteria.

By contrast, in the environmental samples in the vicinity of the airport, we did not find any clear relationship. In the stream, toxicity values found in *L. gibba* were positively correlated to daily precipitation (*r* = 0.96***) and TOC (*r* = 0.95***). At this site, we observed negative correlations (*r* = − 0.88**) between conductivity and TOC (Table 3), indicating that, not the salinity content, but other substances transported to the stream during the precipitation events were responsible for the observed toxicities. It is also possible that conductivity values measured in runoff samples were within the usual range and therefore have no detrimental effect on aquatic life. Our results corroborate findings by other authors who also mentioned the non-suitability of some monospecific bioassays for WWTP effluent toxicity evaluation (Wigh et al., 2016).

pH was negatively correlated with the toxicity values found in *L. gibba* in the lake (*r* = − 0.85*) and with the toxicity values found in *A. fischeri* in the K2 (*r* = − 0.98*). By contrast, pH was positively (*r* = 0.99**) correlated with toxicity values found in *A. fischeri* in the stream (Table 3).

The second PCA component separated the two biotests, indicating that the plant *L. gibba* and the bacteria *A. fischeri*, two organisms from different trophic levels, respond differently to the presence of pollutants in the samples (Fig. 5).

## Conclusions

In this study, we found that the toxicity of the collected airport runoff water samples to the test organisms was mainly influenced by the sampling site (inside and outside the airport). The two organisms investigated in this study are from different trophic levels and have different relationships with the physicochemical parameters of the airport runoff water samples. For sites within the airport, a high correlation between the traditional physicochemical parameters (conductivity and TOC) and toxicity in *L. gibba* was found. However, these correlations were not as clear in environmental water samples taken outside the airport or when *A. fischeri* was used as a bioindicator.

In addition, a pronounced seasonality has been observed, linked to the coldest months (December and January) in which the pavement deicing salts are used, with average inhibition values of 50% in *L. gibba* and 25% in *A. fischeri*, particularly in January.

In contrast, the physicochemical parameters (conductivity and TOC) were influenced by the sampling period and were higher in colder months, while the pH value only differed significantly between sampling points and correlated with the volume of deicer used. The pH value remained stable in the water surfaces outside the airport. Conductivity and TOC did not correlate with the amount of ADAFs applied.

In general, the runoff water samples tested in this study were relatively non-toxic to the test organisms. However, some fertilization effects were found at sites inside the airport that could lead, in the absence of wastewater treatment, to eutrophication processes if the runoff reaches water bodies. Deicing Place showed the lowest toxicity of all locations within the airport, pointing to a lack of toxic compounds in the additives of ADAFs and indicating the influence of other sources, such as salts of PDMs used during the winter months. According to our results, we could not observe any considerable contribution of wind drift in the dispersion of deicing products into nearby surface waters.

The present study has provided data to assess the potential ecotoxicological effects of airport runoff affected by winter operations. Both biotests yielded different results. Therefore, more biotests with other aquatic organisms from different trophic levels should be included. *L. gibba* showed a good response with this type of water samples and could be included in future studies. In our study, we have mainly focused on the physicochemical analyses routinely performed by the airport. However, it would be important to include other physicochemical analyses such as BOD, COD, nitrogen, and phosphorous concentrations to understand the quality of the environment around the airport, as already proposed by Vasquez and Fatta-Kassinos (2013). In addition, long-term evaluations will allow an assessment of chronic toxicity which will provide extensive information of environmental impact of airport runoff.

Actual air traffic is forecast to double in the next 20 years (IATA, 2017), and it is therefore expected that, in addition to a major contribution to climate change, there will also be a significant increase in the environmental impact of airport operations. It is therefore essential to investigate the characterization of the pollutants present in the airport runoff water and their ecotoxicological effects on various aquatic organisms in order to improve the airport’s runoff management and avoid adverse effects in the environment.

## Electronic supplementary material

ESM 1 (DOCX 153 kb)
