# Correction to: Assessing potential aquatic toxicity of airport runoff using physicochemical parameters and *Lemna gibba* and *Aliivibrio fischeri* bioassays

**DOI:** 10.1007/s11356-021-14861-y

**Published:** 2021-07-20

**Authors:** Olga C. Calvo, Gisela Quaglia, Anubhav Mohiley, Maria Cesarini, Andreas Fangmeier

**Affiliations:** 1 Institute of Landscape and Plant Ecology, University of Hohenheim, Ottilie-Zeller-Weg 3, D-70599 Stuttgart, Germany; 2 Department of Environment, Ghent University, Coupure Links 653, B-9000 Ghent, Belgium; 3 Institute of Evolution & Ecology, University of Tübingen, Auf der, Morgenstelle 5, D-72076 Tübingen, Germany

**Correction to: Environmental Science and Pollution Research (2020) 27:40604–40617**

10.1007/s11356-020-09848-0

The article Assessing potential aquatic toxicity of airport runoff using physicochemical parameters and *Lemna gibba* and *Aliivibrio fischeri* bioassays, written by Olga C. Calvo, Gisela Quaglia, Anubhav Mohiley, Maria Cesarini and Andreas Fangmeier, was originally published electronically on the publisher’s internet portal on 15 July 2020 without open access. With the author(s)’ decision to opt for Open Choice the copyright of the article changed on 07 June 2021 to © The Author(s) 2021 and the article is forthwith distributed under a Creative Commons Attribution 4.0 International License, which permits use, sharing, adaptation, distribution and reproduction in any medium or format, as long as you give appropriate credit to the original author(s) and the source, provide a link to the Creative Commons licence, and indicate if changes were made. The images or other third party material in this article are included in the article’s Creative Commons licence, unless indicated otherwise in a credit line to the material. If material is not included in the article’s Creative Commons licence and your intended use is not permitted by statutory regulation or exceeds the permitted use, you will need to obtain permission directly from the copyright holder. To view a copy of this licence, visit http://creativecommons.org/licenses/by/4.0.

The Original article has been corrected.

