# Supplementary material for: Assessing potential aquatic toxicity of airport runoff using physicochemical parameters and Lemna gibba and Aliivibrio fischeri bioassays
Source: Environ Sci Pollut Res Int. 2020 Jul 15;27(32):40604–17. doi: 10.1007/s11356-020-09848-0 (PMC8275521; doi:10.1007/s11356-020-09848-0)
Supplement: Supplementary file 1 — (DOCX 153 kb) [file 11356_2020_9848_MOESM1_ESM.docx]

Assessing potential aquatic toxicity of airport runoff using physicochemical parameters and *Lemna gibba* and *Aliivibrio fischeri* bioassays

# Environmental Science and Pollution Research

O.C. Calvo^1^*, G. Quaglia^1,2^, A. Mohiley^1,3^, M. Cesarini^1^, A. Fangmeier^1^

^1^ *Institute of Landscape and Plant Ecology, University of Hohenheim, August-von-Hartmann- Str. 3, D-70599 Stuttgart, Germany*

^2^ *Department of Environment, Ghent University, Coupure Links 653, B-9000 Ghent, Belgium*

^3^ *Institute of Evolution & Ecology, University of Tübingen, Auf der Morgenstelle 5, D-72076 Tübingen, Germany*

*Corresponding author: O. C. Calvo E-mail: o.calvo@uni-hohenheim.de

Appendix A. Total quantity of ADAFs applied and climatic data during winter 2012-2013.

a) b)


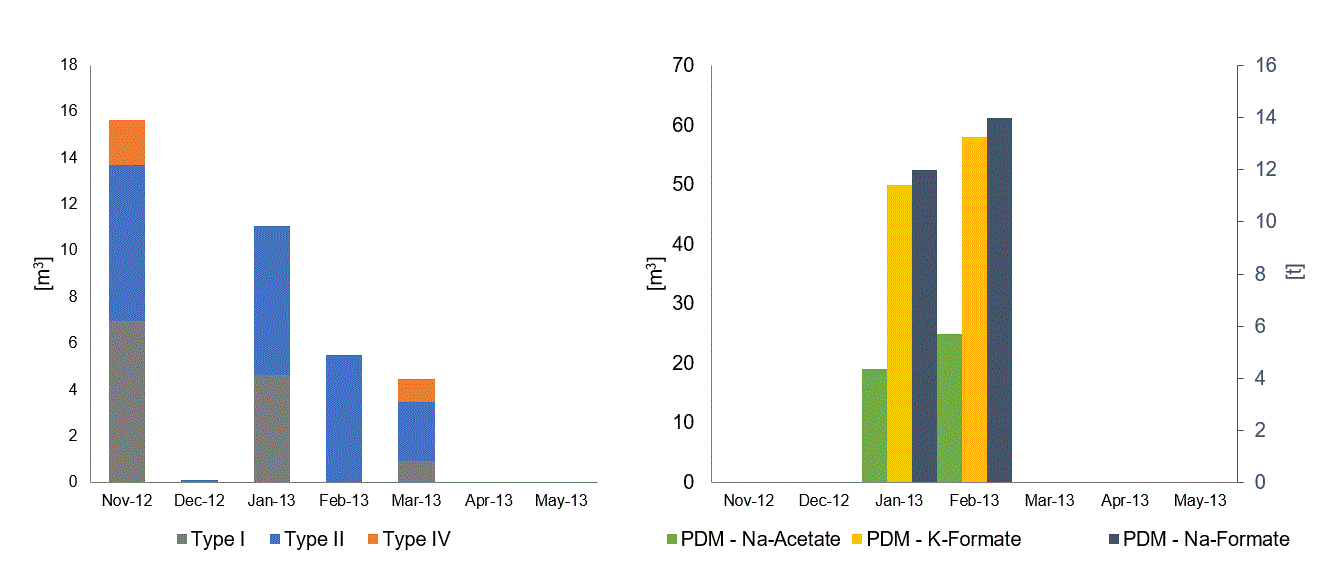


**Fig. A6** a) Total quantity of ADAFs (Type I, Type II and Type IV) applied at the day of sampling at the Deicing Place and b) quantity of liquid PDMs (Na-Ac, K-Fo) and solid PDMs (Na-Fo) applied during winter 2012-2013 (Flughafen Stuttgart, personal communication)

| Date | Temp. | Precipitation |
| --- | --- | --- |
|  | [°C] | [mm] |
| 29.11.2012 | 2.1 | 31.0 |
| 18.12.2012 | 4.0 | 2.8 |
| 22.01.2013 | 0.5 | 1.0 |
| 26.02.2013 | -1.1 | 2.0 |
| 21.03.2013 | 3.7 | 0.8 |
| 23.04.2013 | 12.8 | 0.0 |
| 16.05.2013 | 12.3 | 3.0 |

a) b)

**Fig. A7** Precipitation and atmospheric temperature data from Stuttgart Airport in the winter months 2012-2013. (a) Monthly average and (b) at the day of sampling (LTZ, 2020)

Appendix B. Effective concentrations (EC_50_ / EC_10_) (in volume/volume) of airport runoff collected at different locations in winter months 2012-2013

**Table A4** Effective concentrations (a) EC_50_, (b) EC_10_ of airport runoff collected in different locations in winter months 2012-2013 on *Lemna gibba* frond number (FN) and frond area (FA)

a)

| **Place** | **Month** | **EC_50_ FN** | **SE** | ***p*-value** | **Comment** | **EC_50_ FA** | **SE** | ***p*-value** | **Comment** |
| --- | --- | --- | --- | --- | --- | --- | --- | --- | --- |
| Deicing | Nov | - | - |  | Fertilization | - | - |  | Fertilization |
| Deicing | Dec | - | - |  | Fertilization | - | - |  | Fertilization |
| Deicing | Jan | 80.2 | 22.4 | 1.22E-13 | Quadratic | 73.2 | 18.4 | 1.00E-14 | Quadratic |
| Deicing | Feb | - | - |  | Fertilization | - | - |  | Fertilization |
| Deicing | Mar | - | - |  | Fertilization | - | - |  | Fertilization |
| Deicing | Apr | - | - |  | Fertilization | - | - |  | Fertilization |
| Deicing | May | - | - |  | Not toxic | - | - |  | Not toxic |
| K2 | Dec | 126.1 | 2.7 | 7.54E-12 | Logistic | 79.8 | 4.2 | 1.54E-09 | Logistic |
| K2 | Jan | 99.0 | 3.0 | 2.20E-16 | Cubic | 70.2 | 2.4 | 2.05E-12 | Logistic |
| K2 | Feb | 109.6 | 10.3 | 2.20E-16 | Cubic | 97.6 | 6.7 | 2.20E-16 | Cubic |
| K2 | Mar | 220.4 | 181.7 | 3.05E-13 | Cubic | 146.4 | 77.9 | 4.72E-13 | Cubic |
| K2 | Apr | 240.6 | 45.1 | 6.98E-09 | Lineal | 293.0 | 53.1 | 4.08E-13 | Cubic |
| K3 | Dec | 193.7 | 45.7 | 7.58E-06 | Cubic | 219.4 | 51.0 | 3.61E-06 | Cubic |
| K3 | Jan | 100.6 | 49.5 | 2.26E-09 | Quadratic | 154.8 | 93.0 | 3.76E-06 | Lineal |
| K3 | Feb | 198.7 | 23.0 | 4.94E-03 | Logistic | 193.7 | 31.2 | 1.92E-06 | Cubic |
| K3 | Mar | - | - |  | Fertilization | - | - |  | Fertilization |
| K3 | Apr | - | - |  | Fertilization | - | - |  | Fertilization |
| Lake | Nov | - | - |  | Not toxic | - | - |  | Not toxic |
| Lake | Dec | - | - |  | Not toxic | 222.4 | 18.9 | 1.81E-08 | Logistic |
| Lake | Jan | 180.3 | 6.7 | 4.46E-14 | Logistic | 178.0 | 11.7 | 3.53E-09 | Logistic |
| Lake | Feb | 290.3 | 60.6 | 7.23E-15 | Cubic | 260.3 | 6.9 | 2.20E-16 | Cubic |
| Lake | Mar | 442.9 | 44.3 | 1.44E-08 | Logistic | 266.3 | 7.8 | 3.51E-12 | Cubic |
| Lake | Apr | 319.0 | 40.8 | 7.82E-10 | Lineal | 266.6 | 44.4 | 4.25E-09 | Lineal |
| Lake | May | - | - |  | Not toxic | 478.6 | 143.3 | 2.20E-16 | Cubic |
| Stream | Nov | - | - |  | Not toxic | - | - |  | Not toxic |
| Stream | Dec | - | - |  | Not toxic | - | - |  | Not toxic |
| Stream | Jan | - | - |  | Not toxic | - | - |  | Not toxic |
| Stream | Feb | - | - |  | Not toxic | - | - |  | Not toxic |
| Stream | Mar | - | - |  | Not toxic | - | - |  | Not toxic |
| Stream | Apr | - | - |  | Not toxic | - | - |  | Not toxic |
| Stream | May | - | - |  | Not toxic | - | - |  | Not toxic |

b)

| **Place** | **Month** | **EC_10_ FN** | **SE** | ***p*-value** | **Comment** | **EC_10_ FA** | **SE** | ***p*-value** | **Comment** |
| --- | --- | --- | --- | --- | --- | --- | --- | --- | --- |
| Deicing | Nov | - | - |  | Fertilization | - | - |  | Fertilization |
| Deicing | Dec | - | - |  | Fertilization | - | - |  | Fertilization |
| Deicing | Jan | 13.1 | 13.1 | 1.22E-13 | Quadratic | 12.3 | 11.8 | 1.00E-14 | Quadratic |
| Deicing | Feb | - | - |  | Fertilization | - | - |  | Fertilization |
| Deicing | Mar | 214.9 | 46.1 | 2.14E-05 | Cubic | 258.8 | 34.8 | 1.36E-03 | Cubic |
| Deicing | April | - | - |  | Fertilization | - | - |  | Fertilization |
| Deicing | May | - | - |  | Not toxic | - | - |  | Not toxic |
| K2 | Dec | 29.6 | 1.6 | 7.54E-12 | Logistic | 9.4 | 1.6 | 1.54E-09 | Logistic |
| K2 | Jan | 22.4 | 3.2 | 2.20E-16 | Cubic | 10.4 | 1.1 | 2.05E-12 | Logistic |
| K2 | Feb | 14.3 | 3.6 | 2.20E-16 | Cubic | 12.1 | 1.9 | 2.20E-16 | Cubic |
| K2 | Mar | 16.2 | 5.8 | 3.05E-13 | Cubic | 13.4 | 5.5 | 4.72E-13 | Cubic |
| K2 | April | 48.1 | 40.8 | 6.98E-09 | Lineal | 19.7 | 6.4 | 4.08E-13 | Cubic |
| K3 | Dec | 131.8 | 26.9 | 7.58E-06 | Cubic | 152.1 | 30.0 | 3.61E-06 | Cubic |
| K3 | Jan | 16.5 | 29.5 | 2.26E-09 | Quadratic | 31.0 | 89.9 | 3.76E-06 | Lineal |
| K3 | Feb | 98.7 | 23.2 | 4.94E-03 | Logistic | 123.5 | 22.8 | 1.92E-06 | Cubic |
| K3 | Mar | 292.4 | 62.5 | 4.49E-02 | Quadratic | 302.6 | 35.7 | 3.35E-05 | Quadratic |
| K3 | April | - | - |  | Fertilization | - | - |  | Fertilization |
| Lake | Nov | 83.3 | 30.3 | 6.61E-09 | Quadratic | 206.3 | 46.8 | 1.57E-08 | Lineal |
| Lake | Dec | 24.7 | 5.2 | 2.54E-16 | Quadratic | 17.6 | 3.3 | 1.81E-08 | Logistic |
| Lake | Jan | 16.8 | 1.6 | 4.46E-14 | Logistic | 18.7 | 3.0 | 3.53E-09 | Logistic |
| Lake | Feb | 21.6 | 5.0 | 7.23E-15 | Cubic | 27.4 | 3.8 | 2.20E-16 | Cubic |
| Lake | Mar | 23.4 | 3.1 | 1.44E-08 | Logistic | 24.3 | 8.7 | 3.51E-12 | Cubic |
| Lake | April | 63.8 | 34.6 | 7.82E-10 | Lineal | 53.3 | 39.3 | 4.25E-09 | Lineal |
| Lake | May | 46.6 | 11.1 | 1.64E-13 | Quadratic | 25.3 | 3.2 | 2.20E-16 | Cubic |
| Stream | Nov | 34.9 | 8.6 | 2.42E-11 | Cubic | 240.4 | 138.6 | 1.32E-03 | Lineal |
| Stream | Dec | 67.6 | 31.5 | 7.53E-09 | Cubic | 57.3 | 27.6 | 1.25E-08 | Cubic |
| Stream | Jan | 277.8 | 41.9 | 1.81E-09 | Lineal | 393.1 | 198.3 | 6.38E-03 | Lineal |
| Stream | Feb | 269.2 | 42.0 | 2.05E-09 | Lineal | - | - |  | Not toxic |
| Stream | Mar | 242.3 | 54.4 | 7.30E-08 | Lineal | - | - |  | Not toxic |
| Stream | April | 238.9 | 21.3 | 6.77E-09 | Cubic | 358.3 | 208.8 | 1.07E-02 | Lineal |
| Stream | May | 323.1 | 43.1 | 1.03E-09 | Cubic | 379.2 | 185.8 | 4.55E-03 | Lineal |

**Table A5** Effective concentration (EC_50_ / EC_10_) of airport runoff collected in different locations in winter months 2012-2013 on *Aliivibrio fischeri* bioluminescence

| **Place** | **Month** | **EC_50_ BIO** | **SE** | **Comment** | **EC_10_ BIO** | **SE** | ***p*-value** | **Comment** |
| --- | --- | --- | --- | --- | --- | --- | --- | --- |
| Deicing | Dec | - | - | Not toxic | 33.7 | 12.2 | 1.61E-02 | Cubic |
| Deicing | Jan | - | - | Not toxic | 45.1 | 10.8 | 4.93E-03 | Cubic |
| Deicing | Feb | - | - | Not toxic | 232.8 | 12.4 | 6.56E-03 | Cubic |
| Deicing | Apr | - | - | Not toxic | 225.7 | 0.7 | 1.41E-05 | Cubic |
| K2 | Dec | - | - | Not toxic | 20.4 | 3.9 | 2.88E-03 | Cubic |
| K2 | Jan | - | - | Not toxic | 23.4 | 4.6 | 3.40E-03 | Cubic |
| K2 | Mar | - | - | Not toxic | 44.8 | 2.6 | 2.69E-04 | Cubic |
| K2 | Apr | - | - | Not toxic | 24.5 | 5.2 | 4.20E-03 | Cubic |
| K3 | Dec | - | - | Not toxic | 21.7 | 5.2 | 5.27E-03 | Cubic |
| K3 | Jan | - | - | Not toxic | 20.9 | 5.0 | 4.30E-03 | Cubic |
| K3 | Mar | - | - | Not toxic | 111.4 | 10.8 | 9.03E-06 | Lineal |
| K3 | Apr | - | - | Not toxic | 66.5 | 5.6 | 8.84E-04 | Cubic |
| Lake | Dec | - | - | Not toxic | 142.9 | 54.3 | 2.64E-02 | Lineal |
| Lake | Jan | - | - | Not toxic | 204.3 | 55.7 | 2.23E-02 | Lineal |
| Lake | Mar | - | - | Not toxic | 236.0 | 39.0 | 6.94E-03 | Lineal |
| Lake | Apr | - | - | Not toxic | 184.7 | 21.0 | 1.53E-03 | Lineal |
| Stream | Dec | - | - | Not toxic | 213.5 | 17.3 | 2.25E-03 | Cubic |
| Stream | Jan | - | - | Not toxic | 59.3 | 12.4 | 3.69E-03 | Cubic |
| Stream | Mar | - | - | Not toxic | 63.1 | 17.3 | 5.71E-03 | Cubic |
| Stream | Apr | - | - | Not toxic | 41.6 | 13.8 | 1.06E-02 | Cubic |

Appendix C. Physicochemical properties of ADAFs and runoff samples collected at different locations within an airport during winter months 2012-2013

**Table A6** Physicochemical properties of deicers and pavement deicers (after information provided by the producers)

|  | Typ1 | Typ2 | Typ4 | Sodium Formate |
| --- | --- | --- | --- | --- |
| Propylene glycol content [%] | >80 | >50 | >50 | _ |
| Optimum temperature [°C] | -33 | -36 | -36 | -14 |
| pH | 8.0-9.5 | 7.0-7.5 | 7.0-7.5 | 9.0 |
| Density [g/cm^3^] | 1.04 | 1.04 | 1.04 | 1.92 |
| Colour | Orange | Green | Green | Clear white |
| Algae Toxicity EC_50_ (OECD 201) | >10 g/L | 2.266 mg/L | _ | _ |
| Bacterial Toxicity EC_50_ (ISO 8192) | >10 g/L | 5.2 g/L | 5.2 g/L | _ |
| Company Name | Clariant | Clariant | Clariant | _ |

**Table A7** Physicochemical properties of runoff samples collected at different locations within an airport during winter months 2012-2013

| **Place** | **Month** | **Sampling date** | **TOC [mg L^-1^]** | **pH** | **EC [μS cm^-1^]** |
| --- | --- | --- | --- | --- | --- |
| Deicing | Nov | 29.11.2012 | 96 | 8.0 | 435 |
| Deicing | Dec | 18.12.2012 | 222 | 8.3 | 1025 |
| Deicing | Jan | 22.01.2013 | 800 | 9.4 | 2580 |
| Deicing | Feb | 26.02.2013 | 303 | 8.2 | 1587 |
| Deicing | Mar | 21.03.2013 | 265 | - | - |
| Deicing | Apr | 23.04.2013 | 136 | 7.6 | 1150 |
| Deicing | May | 16.05.2013 | 90 | 7.2 | 803 |
| K2 | Dec | 18.12.2012 | 415 | 8.6 | 2260 |
| K2 | Jan | 22.01.2013 | 410 | 8.9 | 2750 |
| K2 | Feb | 26.02.2013 | 500 | 7.0 | 5250 |
| K2 | Mar | 21.03.2013 | 249 | 9.4 | 3690 |
| K2 | Apr | 23.04.2013 | 41 | 8.4 | 807 |
| K3 | Dec | 18.12.2012 | 1199 | 9.0 | 3440 |
| K3 | Jan | 22.01.2013 | 1655 | 9.6 | 4810 |
| K3 | Feb | 26.02.2013 | 749 | 9.4 | 3430 |
| K3 | Mar | 21.03.2013 | 515 | 9.5 | 1624 |
| K3 | Apr | 23.04.2013 | 289 | 8.9 | 1410 |
| Lake | Nov | 29.11.2012 | 5 | 8.9 | 223 |
| Lake | Dec | 18.12.2012 | 2 | 7.2 | 810 |
| Lake | Jan | 22.01.2013 | 31 | 8.2 | 994 |
| Lake | Feb | 26.02.2013 | 4 | 7.2 | 1240 |
| Lake | Mar | 21.03.2013 | 4 | 7.4 | 774 |
| Lake | Apr | 23.04.2013 | 3 | 7.4 | 1154 |
| Lake | May | 16.05.2013 | 4 | 7.4 | 1036 |
| Stream | Nov | 29.11.2012 | 13 | 8.4 | 426 |
| Stream | Dec | 18.12.2012 | 5 | 7.1 | 850 |
| Stream | Jan | 22.01.2013 | 2 | 7.9 | 837 |
| Stream | Feb | 26.02.2013 | 3 | 7.3 | 834 |
| Stream | Mar | 21.03.2013 | 3 | 8.6 | 897 |
| Stream | Apr | 23.04.2013 | 4 | 8.9 | 800 |
| Stream | May | 16.05.2013 | 5 | 7.5 | 703 |
| H_2_O |  |  | 1 | 7.6 | 188 |

*EC* electrical conductivity, *TOC* total organic carbon

Appendix D. Principal Component Analysis (PCA)


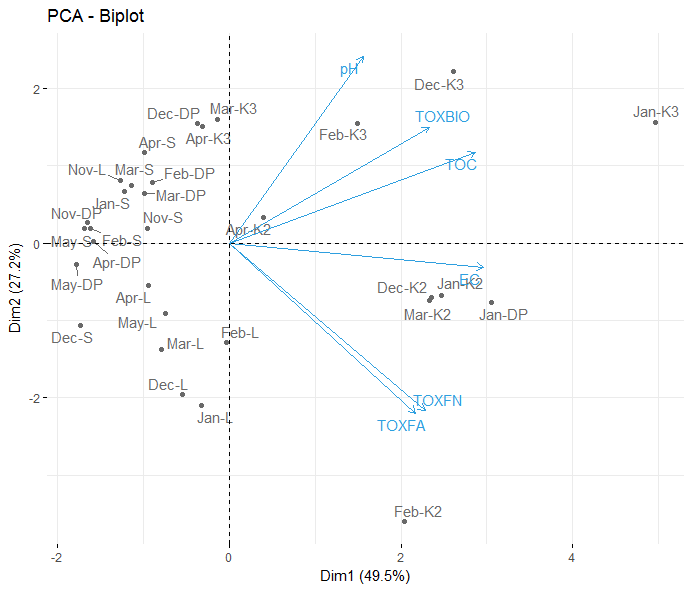


**Fig. A8** PCA-biplot showing the projections of the variables (EC, pH, TOC, TOXBIO, TOXFN, TOXFA according to a) the site and b) the month. Vectors represent the strength and direction of environmental data. TOX toxic value TV10 (FN/FA Frond number/area of *Lemna gibba*. BIO bioluminescence of *Aliivibrio fischeri*), *EC* electrical conductivity, *TOC* total organic carbon

Appendix E. R codes

Packages: drc, investr, lmtest

###EC10 Calculations Frond Number###

#Logistic 4 parameters, upper limit =100, lower limit =0

modsmp=drm(Infnumber ~ Conc, data = smp,

fct = LL.4(fixed = c(NA,0,100,NA), names = c("Slope", "Lower Limit", "Upper Limit", "ED10")))

coeftest(modsmp, vcov = sandwich)

mselect(modsmp, list(LL.3(),LL.4(),LL.5(), W1.3(), W1.4(), W2.4()),linreg = TRUE,icfct = AIC)

plot(residuals(modsmp) ~ fitted(modsmp), main="Residuals vs Fitted")

qqnorm(residuals(modsmp))

qqline(residuals(modsmp))

ED(modsmp,10,interval="delta")

#Lineal

modlineal=lm(Infnumber ~ Conc, data = smp)

invest(modlineal, y0=10, upper = 1000, interval = "Wald")

#quadratic

modquad=lm(Infnumber ~ poly(Conc,2, raw = TRUE), data = smp)

invest(modquad, y0=10, upper = 100, interval = "Wald")

#cubic

modcubic=lm(Infnumber ~ poly(Conc,3, raw = TRUE), data = smp)

invest(modcubic, y0=10, upper = 1000, interval = "Wald")

#Correlations

cor.test(data$EC10FN, data$EC10FA, method="pearson", data=data)
